# Mediating role of depression in the association between alexithymia and nonsuicidal self-injury in a representative sample of adolescents in Taiwan

**DOI:** 10.1186/s13034-022-00477-8

**Published:** 2022-06-15

**Authors:** Wen-Ching Tang, Min-Pei Lin, Jo Yung-Wei Wu, Yueh-Ting Lee, Jianing You

**Affiliations:** 1 Department of Educational Psychology and Counseling, National Taiwan Normal University, No.162, Sec. 1, Heping E. Rd., Da-an District, 106 Taipei City, Taiwan; 2 Good-Day Psychology Clinic, 5F., No. 167, Xialin Rd., South District, 702 Tainan City, Taiwan; 3 Department of Counseling and Guidance, National University of Tainan, No.33, Sec. 2, Shu- Lin St, 700 Tainan, Taiwan; 4 Center for Studies of Psychological Application, Guangdong Key Laboratory of Mental Health and Cognitive Science, Key Laboratory of Brain, Cognition and Education Sciences (South China Normal University), & School of Psychology, Ministry of Education, South China Normal University, 510631 Guangzhou, People’s Republic of China

**Keywords:** Alexithymia, Depression, Nonsuicidal self-injury

## Abstract

**Background:**

Nock’s (2009) integrated theoretical model suggests that specific intrapersonal vulnerability factors caused by distal risk factors contribute to the development of nonsuicidal self-injury (NSSI). Alexithymia and depression have been found to predict NSSI. Based on Nock’s model, alexithymia plays a distal risk factor role to increase the risk of depression—an intrapersonal vulnerability factor—and further increase the risk of NSSI. However, small or unrepresentative samples in past studies limit the generalizability of the results. This study examined the roles of depression and alexithymia in predicting NSSI, as well as the mediating effect of depression in the relation between alexithymia and NSSI in a large representative sample of adolescents in Taiwan.

**Methods:**

Using a cross-sectional study design, a large representative sample of 2,170 senior high school students in Taiwan was assessed by self-report measures of alexithymia, depression, and NSSI. Mediation analyses were performed to examine whether the relation between alexithymia and NSSI was mediated by depression. The questionnaires were administered in classrooms.

**Results:**

Results showed that alexithymia positively predicted NSSI (*β* = 0.23, *p* < .001) and depression can also positively predict NSSI (*β* = 0.41, *p* < .001). Additionally, the association between alexithymia and NSSI was fully mediated by depression.

**Conclusions:**

This study data provided evidence for the mediating role of depression between alexithymia and NSSI, which can be explained by Nock’s (2009) integrated theoretical model. The implications of the findings for future research and intervention were discussed.

## Background

Nonsuicidal self-injury (NSSI) is the voluntary destruction or alteration of body tissue without any suicidal intent, for the purpose that is not socially approved, and the category includes behaviors such as self-cutting, head banging, burning, self-hitting, scratching to the point of bleeding, and interfering with the healing of a wound [1]. NSSI gradually became a growing health problem worldwide and meta-analysis study showed that the lifetime prevalence of NSSI was as high as 17.2% among adolescents, 13.4% among young adults, and 5.5% among adults [2], and many studies found similar prevalence across countries [3, 4]. Moreover, prospective studies indicated that NSSI is a risk factor for suicidal behavior. NSSI is a common problem among adolescents and a highly associated risk factor for later suicidality [5, 6]. Nock et al. [7] found that among adolescents who engage in NSSI, 70% reported a lifetime suicide attempt and 55% reported multiple attempts. NSSI was also included by the DSM-5 in the NSSI disorder as a “condition requiring further study” [8]. Therefore, it is important to investigate risk factors associated with the development and maintenance of NSSI, and to take further preventive action.

Studies in the past have investigated a number of risk factors for NSSI [9–14]. Linehan [15] claims that childhood trauma may lead to the development of poor emotional regulation because children cannot comfort themselves or express their emotions to others. They may develop maladaptive techniques to help them deal with emotions, such as self-harm. Moreover, emotion dysregulation originates from more distal factor like personal predispositions or features [1]. Lacking the ability to identify and regulate emotions in an adaptive way is a feature of the personality trait known as alexithymia. Alexithymia, a personality factor defined as impairment in identifying and describing emotion, and externally oriented thinking [16], may be an important risk factor for NSSI. The relationship between alexithymia and many psychological disorders were found, such as panic disorder and social phobia [17], eating disorder [18], obsessive-compulsive disorder [19], alcohol use disorder [20], major depression [21–28], and personality disorders [29]. Several studies also found the association between alexithymia and NSSI [30–32]. Individuals who have high levels of alexithymia may engage in NSSI to regulate negative feelings and escape from emotional experiences [33]. This link is found among different groups, such as adolescents [10, 11, 34–38], undergraduate students [39–41], and adults [2, 42, 43].

Recently, the association between depression and NSSI has also been highlighted [44]. In several studies, depression is positively correlated with NSSI [13, 45–47]. People who self-harm also tend to suffer from depression [7, 12, 46–49]. Depression brings negative feelings and numbness that is often coped through NSSI [50].

Furthermore, Nock [1] proposed an integrated theoretical model to explain NSSI. NSSI is maintained by intrapersonal vulnerability factors (e.g. aversive emotion and thoughts) or interpersonal vulnerability factors throughout reinforcement processes to regulate affective experiences and social situations, and these vulnerability factors are caused by more distal risk factors (e.g. childhood maltreatment) [1]. Therefore, certain distal risk factors formed particular vulnerability factors, and further resulting in NSSI. Alexithymia is a personality trait that is formed by childhood experience. It arises from emotional development periods in an invalidating or abusive family environment where children learn that it’s inappropriate, ineffective, or dangerous to express and communicate emotions [39]. Adolescents who are not able to describe and identify their feelings may experience the emotional information as overwhelming and confusing, which may increase the risk of depression [51]. Depression is an intrapersonal vulnerability factor that causes negative emotion and cognition. NSSI is engaged to cope with depressive negative feelings. Past research indicated that alexithymia is a personality trait that increases the risk of depression [24]. Patients with depression exhibit higher levels of alexithymia than those with other psychiatric disorders [52, 53]. Furthermore, in longitudinal samples, change in alexithymia can predict change in depression over time [22, 26]. On the other hand, some longitudinal studies found that depression is a predictive factor of NSSI [9, 54].

According to Nock’s [1] integrated theoretical model, distal risk factors are no longer associated with NSSI when vulnerability factors are controlled. Previous studies have provided evidence that depression mediates the relation between alexithymia and self-harm, and suicidal behavior, respectively [36, 55, 56]. Hintikka et al. [56] claimed that the association between alexithymia and suicidal ideation was mostly explained by depression. Lambert and de man [36] indicated that self-mutilation was used to regulate depression among adolescent girls with alexithymia. Garisch and Wilson [55] found that depression mediated the relation between alexithymia and deliberate self-harm. Although past studies have found that depression mediates the relationship between alexithymia and self-harm, however, most of them rely on small or unrepresentative samples which limits the generalizability of the results. Therefore, the present study incorporated a large sample in Taiwan to overcome this limitation. In addition, the exploration of a different culture, Taiwan, can also increase the generalizability of findings. Furthermore, past studies target self-harm behavior and suicidal behavior. NSSI is the voluntary destruction or alteration of body issue without any suicidal intent, which is distinguished from suicidal behavior. Given these research gaps, the present study examined the associations among alexithymia, depression, and NSSI in a large representative sample of adolescents in Taiwan. We developed a mediational pathway based on Nock’s integrated theoretical model [1], and proposed a hypothesis: It was expected that depression would mediate the relation between alexithymia and NSSI.

## Methods

### Participants and procedure

This study used both stratified and cluster sampling to recruit participants from senior high schools throughout Taiwan. A representative sample of senior high school student was acquired from the population in Taiwan. Based on the data from the Department of Statistics Ministry of Education’s Department of Statistics in Taiwan, school type (regular high school or vocational high school) was used as a stratum, and classes as the cluster. A total of 2253 senior high school students were recruited to join in the study. After culling invalidated questionnaires and deleting the participants with missing values, the final sample of 2170 students participated (Mage = 16.83, SD = 0.38 years; female = 1127, male = 1035, missing = 8), resulting in a response rate of 96.32%.

The study protocol was approved by the review board of the Ministry of Science and Technology in Taiwan. All study procedures and ethical aspects were followed. The purpose and content of the study were clearly stated to the school authorities and teachers prior to the assessment. Consent forms were collected from legal representatives in the schools before administering the questionnaires. Consent forms were also provided to students’ guardians informing the purpose and content of this study. Emphasis was given that participation was voluntary. Teachers had to highlight the confidentiality of the surveys and collect the written consent forms from the participants. The questionnaires were administered in classrooms.

### Measures

Alexithymia. Alexithymia was assessed by the 20-Item Toronto alexithymia scale (TAS-20) [16]. It is a self-report questionnaire containing 20 items on a 5-point Likert scale, ranging from 0 (strongly disagree) to 5 (strongly agree). The TAS-20 included 3 subscales: Difficulty Describing Feelings (5 items), Difficulty Identifying Feelings (7 items), and Externally-Oriented Thinking (8 items). The TAS-20 exhibited good internal consistency, test-retest reliability, and factorial validity [16, 57]. Taylor [58] reviewed studies that evaluated the reliability and factorial validity of the 20-item Toronto Scale (TAS-20) in different languages and cultures including Taiwan. There is strong support for the generalizability of the three-factor structure of the scale across languages and cultures. Moreover, previous studies also found that the Taiwanese version of the TAS-20 showed good internal consistency (α = 0.84) and factorial validity [58]. In the present study, the TAS-20 also showed good internal consistency (α = 0.84).

Depression. Participants’ depression levels were assessed by the 26-item Ko’s Depression Inventory (KDI), and it was constructed in references from various depression scales [59]. KDI contains four subscales: Affective (7 items), Physiological (9 items), Behavior (2 items), and Cognitive (8 items) [60]. This scale was positively related to the Depression subscale of the Symptom Checklist-90-R (*r* = .73), which demonstrated the validity of KDI among adolescents in Taiwan [59]. The Cronbach’s alpha for the KDI in this study was 0.89.

Nonsuicidal self-injury. The NSSI inventory consists of 12 different methods of NSSI which were selected from the Deliberate Self-Harm Inventory [61]. According to Lloyd-Richardson et al. [62], NSSI could be distinguished between severe NSSI methods (7 items) and minor NSSI methods (5 items). Moreover, the 12 items were the most common NSSI behaviors in previous studies among Chinese adolescents [14, 63–66]. Participants were asked, “Have you engaged in the following behaviors to deliberately harm yourself without suicidal intent in the past year?” The inventory contained 12 items each measured on a 6-point scale, ranging from 0 to 5 (0 = never, 1 = once, 2 = twice, 3 = three times, 4 = four times, 5 = five times or more). We calculated a total frequency of NSSI by summing up scores of the 12 NSSI items. This scale has shown sufficient concurrent and overtime validity via its relationships to other psychopathology measures [67]. In the current study, this scale has a Cronbach’s alpha value of 0.84.

### Data analyses

Data were analyzed using SPSS for Windows version 18.0 (SPSS Inc., Chicago, IL USA) for computing descriptive statistics and Pearson correlations. Mediation analyses were performed to examine whether the relationship between alexithymia and NSSI was mediated by depression. The mediation model is tested by structural equation modeling (SEM) analyses with the robust maximum-likelihood method by AMOS 18.0 (SPSS Inc., Chicago, IL USA), and we used Baron & Kenny’s [68] step process, Sobel test, and bootstrap analyses to test the mediation model, respectively [69, 70].

## Results

### Descriptive statistics

436 adolescents reported having engaged NSSI at least once. 171 adolescents reported using only one method (40.9%). 247 adolescents reported using multiple methods (59.1%). Additionally, 99 adolescents engaged in NSSI only once (24%), while 319 adolescents have performed NSSI twice or more in the past (76%).

### Correlations

All means, standard deviations, and correlations of the dependent and independent variables are provided in Table 1. Findings showed that alexithymia was significantly and positively correlated with depression and NSSI, respectively. Moreover, depression and NSSI were positively correlated with each other.

**Table 1:** The correlations among variables

Variables	1		2	3	4	5	6	7	8	9	10
1. Alexithymia	–										
2. Difficulty describing emotions	0.87^***^		—								
3. Difficulty identifying emotions	0.90^***^		0.74^***^	–							
4. Externally oriented thinking	0.56^**^		0.30^***^	0.20^***^	–						
5. Depression	0.48^***^		0.40^***^	0.53^***^	0.12^***^	–					
6. Affective	0.44^***^		0.37^***^	0.50^***^	0.09^***^	0.87^***^	–				
7. Physiological	0.33^***^		0.25^***^	0.36^***^	0.11^***^	0.81^***^	0.57^***^	–			
8. Behavior	0.33^***^		0.29^***^	0.37^***^	0.06^**^	0.69^***^	0.54^***^	0.51^***^	–		
9. Cognitive	0.47^***^		0.42^***^	0.51^***^	0.11^***^	0.90^***^	0.71^***^	0.58^***^	0.61^***^	–	
10. NSSI frequency	0.19^***^		0.14^***^	0.21^***^	0.07^***^	0.38^***^	0.30^***^	0.32^***^	0.25^***^	0.36^***^	–
*M*	53.29		13.81	18.36	21.12	12.15	4.06	3.37	0.54	4.19	1.22
*SD* *Skewness* *Kurtosis*	10.85 − 0.16 − 0.11		3.66 − 0.08 − 0.06	6.24 0.14 − 0.36	3.73 − 0.55 0.39	9.28 1.21 1.74	3.19 0.96 0.88	3.02 1.56 3.62	0.85 1.85 3.74	3.84 1.26 1.77	4.06 5.77 41.97

^**^p < .01; ^***^p < .001

### The mediating role of depression

**Fig. 1:**
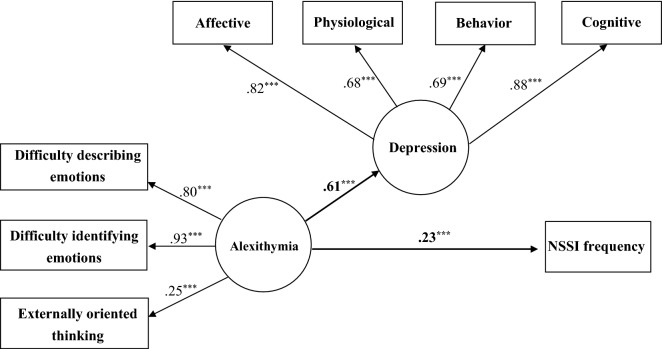
Mediational model 1. Chi-square: 376.437^***^ (df: 19). GFI: 0.956; CFI: 0.944; IFI: 0.944; NFI: 0.942; NNFI: 0.918; RMSEA: 0.096; SRMR: 0.070; ^***^p < .001

**Fig. 2:**
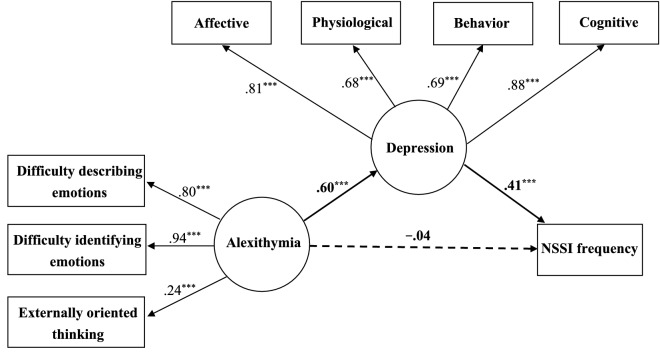
Mediational model 2. Chi-square: 195.366^***^ (df: 18). GFI: 0.977; CFI: 0.972; IFI: 0.972; NFI: 0.970; NNFI: 0.957; RMSEA: 0.070; SRMR: 0.031; ^***^p < .001

We used the SEM by the robust maximum-likelihood method to examine the hypothesized mediational model and deleted the participants with missing values from the model for bootstrap analyses. Consequently, 131 participants were deleted from the mediation analysis, yielding a final sample of 2,039 participants included in the SEM analysis. According to Baron & Kenny [68], testing the mediation effect requires significant correlations between (1) the independent and dependent variables; (2) the independent and proposed mediating variable. Besides, there must have been a significant reduction in the direct path from the independent to the dependent variables when the analysis included the indirect (mediated) effects. Thus, the model was used to test the mediating hypothesis of “Alexithymia—Depression—NSSI”. As shown in Fig. 1, alexithymia had a significant effect on depression and NSSI, respectively. Furthermore, the effect of alexithymia on NSSI decreased from 0.23 (*p* < .001) to − 0.04 (*p* = .213) when the analysis included depression (Fig. 2). Moreover, the Sobel test [69] indicated that the mediated effect was significant for “Alexithymia—Depression—NSSI” (*z* = 8.015, *p* < .001), demonstrating a full mediating effect from alexithymia to NSSI through depression. Results also showed that the fit indices in the models were 0.977 on the GFI, 0.972 on the CFI, 0.972 on the IFI, 0.970 on the NFI, 0.957 on the NNFI, 0.070 on the RMSEA, and 0.031 on the SRMR. The measurement model demonstrated a good fit [71]; the overall fit of the mediational model was adequate and able to explain 14.8% of the variance.

This study also used bootstrapping analyses to examine the indirect effect of alexithymia on NSSI via depression. Bootstrapping method does not presume normality and also has higher power and better Type I error control than other mediation analyses. Meanwhile, bootstrapping provides a more reliable estimate of indirect effects. In order to prove the significance of the mediation model, the confidence interval must not include zero [70, 72]. 5000 bootstrap samples from the data set were created [73], and a bias-corrected 90% confidence interval for the estimates of the indirect effect was used. Results indicated that the mean indirect (nonstandardized) effect of alexithymia on NSSI via depression was 1.095, and the bias-corrected 90% confidence interval was between 0.781 and 1.517, which did not include zero. Thus, the indirect effect was statistically significant (*p* < .001). In addition, the mean direct (nonstandardized) effect of alexithymia on NSSI was − 0.164, and the bias-corrected 90% confidence interval was between − 0.411 and 0.057, which included zero, and therefore the direct effect was statistically non-significant (*p* = .231). The results of the mediation analysis are presented in Table 2. The full mediation model was confirmed.

**Table 2:** Mediating effect of depression between alexithymia and NSSI

	Path	Effect value (nonstandardized)	Boot CI Upper	Boot CI Lower
Indirect effect	Alexithymia-Depression-NSSI	1.095^***^	1.517	0.781
Direct effect	Alexithymia-NSSI	− 0.164	0.057	− 0.411

^***^p < .001

## Discussion

NSSI appears to be an important problem for adolescents. We conduct this study based on Nock’s integrated theoretical model, which infers that NSSI is maintained by vulnerability factors and these factors are caused by distal factors. The present study examined the relationship between alexithymia, depression, and NSSI. The current study found that alexithymia predicted an increase in depression, which played a full mediation role between alexithymia and NSSI of adolescents.

Our results showed that the relation between alexithymia and NSSI was fully mediated by depression, which is similar to the results of previous literature [36, 55, 56]. Hintikka et al. [56] claimed that the association between alexithymia and suicidal ideation was mostly explained by depression. People with alexithymia have trouble thinking about, understanding, or talking about distressing feelings, which may result in depression. Under such circumstances, suicidal ideations often occur when individuals are faced with stressful life events. Lambert and de man [36] investigated fifteen adolescent girls with histories of self-mutilation and eighteen adolescent girls without such a history. Among adolescent girls with alexithymia, self-mutilation was engaged to regulate depression. Garisch and Wilson [55] examined 325 students and found that among bullied adolescents, depression mediated the relationship between alexithymia and deliberate self-harm. Among bullied adolescents, alexithymia increased their vulnerability to self-harm when they also suffer from depression. It is not possible to infer a direct causal relationship between alexithymia and NSSI but that a combination of low mood and poor emotional regulation increases the risk of NSSI. Nock’s integrated theoretical model [1] indicated that distal factor causes vulnerability factor, and further increases the risk of NSSI. Moreover, when vulnerability factors are controlled, distal factors are no longer associated with NSSI. In our study, the effect of alexithymia on NSSI decreased from 0.23 (*p* < .001) to − 0.04 (*p* = .213) when the analysis included depression (Fig. 2). Alexithymia, which plays the role as a distal factor, increases the risk of depression and further the risk of NSSI. Adolescents with alexithymia have trouble recognizing and expressing their emotions, they may suppress or escape from their feelings and become more overwhelmed, which causes depression. Accordingly, they are unable to face their depression and in turn engage in NSSI to deal with their negative emotions.

Consistent with previous research, NSSI can be significantly and positively predicted by alexithymia [10, 74]. Having a restricted ability to identify, understand, and express an emotional state may increase the risk of NSSI [75]. Individuals who have difficulty being aware of their emotional states may be less able to identify and implement strategies for tolerating distress or solving underlying problems, and thus engage in NSSI to alleviate aversive arousal in the short term [76]. For the sake of difficulties mentalizing and expressing their feelings, NSSI individuals use their bodies to cope with their psychological pain. Alexithymia causes NSSI because it creates negative emotions that are difficult to deal with. However, among the three factors of Alexithymia, “externally oriented thinking” has a low factor loading, which corresponds with past studies [31, 42]. A possible explanation is that because of the distinction between the construct of “externally oriented thinking” and the other two factors [77], the factor loading of “externally oriented thinking” was relatively low.

Adolescents who are not able to describe and identify their feelings may experience the emotional stimulus as overwhelming and confusing, which may increase the risk of depression. Our research found that alexithymia can positively predict depression, which is in accordance with past studies [22, 51, 78]. Many researches indicated that alexithymia is common among patients with depression. The severity of depression is strongly associated with alexithymic features [22]. Individuals suffering from depression may suppress their emotions to cope with their symptoms and thus demonstrate more difficulties in subjectively identifying and describing their emotions [53]. Adolescents who have difficulties mentalizing their feelings may suppress aversive thoughts and feelings which is central to depression.

Our study also found that NSSI was predicted by depression. Past studies indicated that people engage in NSSI as a way to cope with negative emotions [79], in order to reduce the intensity of negative emotions associated with depression [62, 80–83]. Depression includes both negative emotions and numbness that NSSI may serve to alleviate, although the relief is short-lived and the symptoms of depression are likely to reoccur [50]. Thus, individuals may become more depressed and engage in NSSI in an attempt to relieve depression [1, 79], and reinforce its continuation [83, 84]. Depressive adolescents tend to be exposed to aversive thoughts and feelings, and NSSI is therefore used to cope with these thoughts and feelings. Alexithymia is a relatively distal risk factor, and therefore, we can target on alleviating depression to lower the severity of NSSI among adolescents.

Some limitations need to be proposed. First, as the results of the current study are based on cross-sectional data, our suggested model needs to be further investigated in a longitudinal study to clarify the relationships. Secondly, all information from our study was obtained from self-reported questionnaires, which might result in biases in the data through the social desirability of the respondent. Future studies may consider incorporating multiple-method assessments to acquire a richer understanding of NSSI. Finally, the high-frequency NSSI group was coded at “5 times or more”. The variable of the current study may be continuous or not. Therefore, future studies can ask participants to provide direct answers of how many times they have engaged in NSSI. The consequence may be clearer.

The present study examined the associations among alexithymia, depression, and NSSI in a large representative sample of adolescents in Taiwan, increasing the culture and sample generalizability of findings. NSSI is the voluntary destruction or alteration of body issue without any suicidal intent, which is distinguished from suicidal behavior. The present study developed the mediational pathway from Nock’s model, and the mediational pathway has partly confirmed the Nock’s model, which help us reach a better understanding in the relationship of alexithymia, depression and NSSI.

## Conclusions

We conduct this study based on Nock’s integrated theoretical model, in which NSSI is maintained by intrapersonal vulnerability factors and these vulnerability factors are caused by more distal risk factors. The current study examined the relationship between alexithymia, depression, and NSSI in a representative sample of adolescents in Taiwan. The goal of this study is to determine whether the relationship between alexithymia and NSSI is mediated by depression. Our results indicated that both alexithymia and depression positively predicted NSSI. In addition, the relationship between alexithymia and NSSI was fully mediated by depression. The result is in accordance with Nock’s integrated theoretical model, indicating that NSSI is maintained by these reinforcement processes to regulate depression, and alexithymia plays a distal risk factor role. The results from our study provide evidence in explaining how depression fully mediated the relationship between alexithymia and NSSI. Our results might help mental health organizations and educational agencies to develop NSSI prevention programs aimed at the senior high school population.

## Data Availability

The data and materials that support the findings of this study are available from the corresponding author upon reasonable request.

## Abbreviations

NNSI: Nonsuicidal self-injury
TAS-20: The 20-Item Toronto alexithymia scale
KDI: The 26-item Ko’s Depression Inventory
SEM: Structural equation modeling
